# The Role of Anxiety and Depressive Symptoms on Nurse-Patient Mutuality in Older Adults with Chronic Illnesses

**DOI:** 10.1093/geroni/igaf122.4216

**Published:** 2025-12-31

**Authors:** Silvia Cilluffo, Karen Lyons, Bassola Barbara, Rosario Caruso, Stefano Terzoni, Maura Lusignani

**Affiliations:** University of Milan, Milan, Lombardia, Italy; Boston College, Chestnut Hill, Massachusetts, United States; ASST GRANDE OSPEDALE METROPOLITANO NIGUARDA, MILAN, Lombardia, Italy; University of Milan, Milano, Lombardia, Italy; Università degli Studi di Milano, Milan, Lombardia, Italy; University of Milan, Milan, Lombardia, Italy

## Abstract

The interactive process (mutuality) between older patients with multiple chronic illnesses and their nurses has received little investigation, despite the therapeutic nature of the relationship. Even less is known about the role of the older patient’s mental health on the perception of this therapeutic relationship. This paper reports novel findings from recently available data from a multi-center study. The sample of 145 patients (Mage= 67.5, ±15.4) were recruited from hospitals across Italy. Patients had to be older adults with 2 or more chronic illnesses. Nurse-patient mutuality was assessed using a validated measure developed by this team consisting of three dimensions: developing rapport/going beyond, becoming a point of reference, and shared decision-making/care. Findings from multiple regression analyses found that both anxiety and depressive symptoms were significantly associated with the patient’s interaction with their nurse. Higher levels of anxiety were significantly positively associated with the developing rapport/going beyond dimension (e.g., trusting the nurse, empathic interactions) (p < .01), and point of reference dimension (e.g., asking the nurse for advice) (p < .001). In contrast, higher levels of depressive symptoms were negatively associated with the developing rapport/going beyond dimension (p< .01) and point of reference dimension (p < .01). Neither anxiety or depressive symptoms were significantly associated with the shared decision-making/care Discussion will focus on the role of mental health when older patients with multiple chronic illnesses interact with their nurse and implications for optimizing the nurse-patient relationship to support better health outcomes.

